# The interconnection between social media addiction, alexithymia and empathy in medical students

**DOI:** 10.3389/fpsyt.2024.1467246

**Published:** 2024-09-19

**Authors:** Sorin Ursoniu, Ana-Cristina Bredicean, Costela Lacrimioara Serban, Ioana Rivis, Adina Bucur, Ion Papava, Catalina Giurgi-Oncu

**Affiliations:** ^1^ Department of Functional Sciences, Discipline of Public Health, Center for Translational Research and Systems Medicine, “Victor Babes” University of Medicine and Pharmacy, Timisoara, Romania; ^2^ Department of Neuroscience, Discipline of Psychiatry, Center for Cognitive Research in Neuropsychiatric Pathology (NeuroPsy-Cog), “Victor Babes” University of Medicine and Pharmacy, Timisoara, Romania; ^3^ Psychiatry Compartment, “Dr. Victor Popescu” Emergency Military Clinical Hospital, Timisoara, Romania

**Keywords:** social media addiction, medical students, empathy, smartphone addiction, alexithymia

## Abstract

**Introduction:**

This study explores whether high alexithymia values correlate with low levels of empathy, while also trying to identify potential connections with social media addiction.

**Methods:**

We hypothesized that alexithymia mediates the relationship between social media addiction and empathy levels in a sample of undergraduate students. The study population consisted of 649 medical students in the 4th/5th/6th University year, recruited between March and May 2021. For this assessment, we employed three psychometric instruments: the Toronto Empathy Questionnaire (TEQ), the Social Media Addiction Scale-Student Form (SMAS-SF), and the Toronto Alexithymia Scale (TAS-20). A pathway analysis investigated alexithymia as a mediator between social media addiction and the degree of empathy in medical undergraduates. Sobel’s test and the Baron and Kenny approach were used for testing mediation.

**Results:**

The TEQ total mean score was 48.76 ± 5.65, while the TAS-20 total mean score was 47.71 ± 11.49. Further analysis of the TAS-20 scale scores showed that 21.42% of students had possible alexithymia, while 14.02% had clear alexithymia. The SMAS-SF total mean score was 73.20 ± 14.59. None of the students reported levels consistent with major social media addiction. The mediated effect of the TAS-20 is about 1.3 times larger than the direct effect of the SMAS-SF on TEQ.

**Discussion:**

We found a significant negative correlation between empathy and alexithymia in medical students. Alexithymia was a mediator between social media addiction and empathy. Therefore, we recommend further efforts to identify potential levels of alexithymia in medical students, in order to successfully develop tailored interventions aimed at increasing their emotional awareness.

## 1 Introduction

Alexithymia represents an inability to describe one’s emotional states. Several studies point out that alexithymia disrupts the ability to identify feelings of others (1). From an etymological point of view, the word comes from Greek: a = lack, lexis = word, and thymos = mood or emotion. Thus, it can be translated as the inability to read and express emotions. The term was coined during the 1970s by two psycho-therapists (Peter E. Sifneos and John C. Nemiah), looking to summarize symptoms they had noticed in their patients suffering from psychosomatic illnesses (2–4). More recently, there has been a growing scientific interest in the way alexithymia affects interpersonal relationships, with some authors hypothesizing that alexithymic individuals have difficulty interacting with others, since alexithymia correlates with emotional skills-related problems, such as difficulties in building and maintaining interpersonal relationships, smaller social networks, and reduced social skills (5, 6).

Since alexithymia is frequently linked to depressive and anxious states of mind, it is likely that people with alexithymia have trouble controlling their negative emotions (1, 7). Alexithymia along with high impulsivity, has been correlated with problematic internet use, consequently raising the theory that people who struggle to identify and communicate their emotions are more inclined to use online video games to escape negative feelings (8). Social media has increased over the past years and has been proven to be used as a maladaptative coping mechanism (9). The use of emotion- and avoidance-oriented coping strategies is favorably correlated with alexithymia, as assessed by the TAS-20 in teenage samples, and negatively correlated with task-oriented coping strategies (10). Di Blasi et al. view the emotional challenges associated with impulsivity and alexithymic features as specific elements that constitute an emotion dysregulation process (11). In alexithymic individuals, restricted and intolerant attitudes toward their own short-comings and limitations, as well as a failure to recognize themselves as a part of the larger human condition, go alongside a constricted awareness of emotions in self and others (12). Therefore, maladaptive coping mechanisms, such as drinking and negative emotional states, like despair, anxiety, and stress can be frequently linked to alexithymia (13).

The prevalence of alexithymia in the general population is estimated to be around 10% (14–16). Some researchers established that this deficiency exists in both healthy and unhealthy individuals (17), characterized by difficulties in identifying, analyzing, and ex-pressing emotions, as well as involving certain restrictions, in terms of externally oriented thinking and imagination. A 5-year follow-up study in the general population has indicated that alexithymia can essentially be considered a stable personality trait (18). There are different theories regarding the emergence of alexithymia, with some studies showing that childhood trauma is a substantial contributing element (19). Some authors suggest that childhood trauma, including emotional abuse and severe emotional and physical neglect, can predict the emergence of alexithymia in later life (20).

According to the research examining the relationship between alexithymia, social media use, and smartphone addiction, there was a strong correlation between alexithymia and the severity of smartphone use (21).

The ability to identify and control one’s own emotions as well as those of others is known as emotional intelligence (EI). On the other hand, empathy is the capacity to comprehend the feelings of others, and alexithymia is the inability to feel and communicate emotions verbally. Emotional intelligence is a crucial prerequisite for success in the medical field (22).

Empathy has a multidimensional character, comprising cognitive and emotional dimensions. It includes the ability to perceive the perspective of others, correctly identify their subjective reality, and have appropriate affective responses that follow the perception of the emotional states of those around us (23).

Empathy has two components: affective, which comprises the individuals’ capacity to feel what others feel; cognitive, which is the ability to identify, interpret and understand the mental states of others, which involves perspective-taking (24, 25). Empathy helps create and maintain these social connections, allowing people to understand, share and respond accordingly to other people emotional states (26).

In healthcare, the professional-patient relationship is primarily based on empathy, as this can help build trust and improve communication, thus creating a safe environment to explore existing possibilities and make the best medical decisions (27, 28). Alternatively, a healthcare professional showing high levels of empathy has been shown to help reduce patients’ stress levels, as well as their anxiety and depression, as well as improve their prognosis (29, 30).

As medical professionals on the frontline of dealing and acting immediately and without fail for the most vulnerable, doctors must be highly specialized. On the path to-ward developing their medical career, medical students must spend significantly more time acquiring the required professional knowledge. Research suggests that this particular area of expertise involves significantly higher academic pressure than other disciplines (31). Keeping this in mind, it becomes somewhat inevitable to logically infer that, as a direct cause of these inherent and long-term pressures, medical students are at a high risk of developing mental health issues, such as burnout, anxiety or depression (32), as alexithymia is a recognized risk factor for these mental health difficulties, particularly in health science students (33). These facts indicate that alexithymia may negatively impact the health professional-patient relationship, starting early on and notable in medical students’ personal and professional lives (34).

In recent years, socializing via the Internet has become an increasingly integral part of young adults’ lives. Social networking sites are online communication tools that allow users to create a public or private profile to interact with others. Although they have helped people connect in new and innovative ways, several researchers have pointed out that excessive use of social networks has negative health consequences, including structural and functional changes in brain regions involved in emotional processing, attention, decision-making, and cognitive control.

Pertaining to our subject at hand, there are differing views regarding the correlation between social media use and empathy. Various studies have noted that empathy is negatively correlated to social media addiction (35), as, according to Dailey (36), empathetic individuals are less likely to develop a social media addiction.

However, certain studies have suggested that this is an indirect connection (37, 38), some authors have demonstrated the opposite (39, 40), while others have highlighted mostly a neutral relationship between the two (41).

This study aimed to explore if high values of alexithymia in medical students correlate with low values of empathy, and to identify any potential connections with social media addiction. We hypothesized that alexithymia mediates the relationship between social media addiction and the degree of empathy in undergraduate medical students, all of which are elements that may influence future medical careers, since these factors influence how we relate to those around us.

## 2 Materials and methods

The study population consisted of 649 medical students in their 4th, 5th, or 6th year of studies, recruited between March and May 2021. The sample is part of one designed for a survey with larger scope, aiming to identify the relationship between the use of social networking and individual Theory of Mind (ToM), empathy and alexithymia levels in undergraduate students.

### 2.1 Socio-demographic features of participants

The mean age of participating students was 23.45 ± 1.30 years. Of all the participants, 79.66% were female, 38.83% were fourth‐year students, 26.35% were fifth‐year students, and 34.82% were sixth‐year students. The socio-demographic characteristics of the sample are shown in **Table 1**

**Table 1:** Socio-demographic features of the student sample (N = 649).

Features		n	%
Gender	Female	517	79.66
	Male	132	20.34
Age category	21–22 years old	173	26.66
	23-24 years old	357	55.01
	25+ years old	119	18.33
Year of Study	4th	252	38.83
	5th	171	26.35
	6th	226	34.82

The study was conducted according to the guidelines of the Declaration of Helsinki, and approved by the Research Ethics Committee of the “Victor Babes” University of Medicine and Pharmacy of Timisoara, Romania (protocol No. 15/20.03.2020).

Informed consent was obtained from all subjects involved in the study.

### 2.2 Instruments

We used several instruments for this assessment. The Toronto Empathy Questionnaire (TEQ) was priorly validated in Romanian (42) and measures the empathy level as a uni-dimensional instrument, consisting of 16 items, with a 5-point Likert scale-type answer: never, rarely, sometimes, often, always, and with scores ranging from 0 to 4. Total scores range from a minimum of 16 to 64 points. The validation study for TEQ in Romanian (42) reported a Cronbach’s alpha of 0.727 and an ICC of 0.776. The validation of TEQ was conducted using the same sample as in the current study.

The Social Media Addiction Scale-Student Form (SMAS-SF) was developed by Sahin et al. (43) and was previously validated in Romanian (44). The questionnaire consists of 29 items with a 5-point Likert scale, from “totally disagree” (1 point), “disagree” (2 points), “neither agree nor disagree” (3 points), “agree” (4 points), “totally agree” (5 points). The total score ranges from a minimum of 29 to a maximum of 145. The total score can be interpreted as: no addiction (≤ 58 points), mild addiction (59-87 points), moderate addiction (88-116 points), and severe addiction (≥ 117 points). The validation of the SMAS-SF in Romanian (44) demonstrated a Cronbach’s alpha of 0.817 and an ICC of 0.829 and used the same sample as in the current study.

The Toronto Alexithymia Scale (TAS-20) was developed by Bagby et al. (45) and consists of 20 items measuring the difficulty in identifying and describing emotions. Alexithymia, as measured by this instrument, is characterized by three factors. The first, entitled “difficulties in identifying feelings” (DIF), and the second factor, entitled “difficulties in describing feelings” (DDF), refer to emotional awareness and expression. They might, therefore, be considered as “affect-related”. The third factor, entitled “externally-oriented thinking” (EOT), refers to a specific tendency to deal with simple themes and to avoid affective thinking. Possible answers are quantified on a 5-point Likert scale, from “strongly disagree” (1 point), “disagree” (2 points), “neither agree nor disagree” (3 points), “agree” (4 points), “strongly agree” (5 points). We employed the recently validated Romanian version (46) for this study. Total scores ranged from 20 points, as a minimum, to 100 points as a maximum. Scores were classified as non-alexithymia (≤51 points), possible alexithymia (52‐60 points), and alexithymia (≥61 points) (47). The validation study in Romanian (Morariu, 2013) reported a Cronbach’s alpha of 0.83. For the TAS-20, the Cronbach’s alpha in the current sample was 0.749.

Besides the specific questionnaires mentioned above, the survey also included demographic questions, such as gender, age, year of study, and final average grade in the previous academic year.

The survey was hosted on a platform and could be accessed using a Google Play application (android and iOS) or a desktop version (https://timsonet.ro). Students could access the survey using a series of alphanumeric codes, randomly generated to assure anonymity.

The final database was imported to the Stata program version 16.1 (StataCorp, College Station, Texas, USA). The categorical variables are represented as absolute and relative frequencies, and continuous variables are presented as mean and standard deviation (SD). A p-value < 0.05 was considered statistically significant. Since the data were not normally distributed, we used the Mann-Whitney test to test for differences between males and females for total scores. The degree of correlation between different questionnaires was test-ed with the Pearson point product correlation. Using structural equation modeling, we created a pathway analysis investigating alexithymia as a mediator between social media addiction and the degree of empathy in medical undergraduates.

The mediation model was analyzed using sgmediation2 command in Stata. For the best test of mediation effect, the bootstrapping procedure to measure indirect effect was carried out and 95% confidence intervals were estimated. The number of bootstrap samples was 5000.

## 3 Results

### 3.1 The participants’ scales’ mean scores

The study’s findings showed that the TEQ total mean score was 48.76 ± 5.65, with scores ranging from 18 to 63. The TAS total mean score was 47.71 ± 11.49, with scores ranging from 23 to 83 (**Table 2**). Further analysis of the TAS scale scores showed that 21.42% of the students had possible alexithymia, and 14.02% had clear alexithymia. The SMAS-SF total mean score was 73.20 ± 14.59, with scores ranging from 31 to 115. A detailed analysis of the SMAS-SF scale scores indicated that as much as 67.18% of the students had a mild addiction, and 15.72% had a moderate addiction. None of the students presented levels consistent with major social media addiction. The use of the SMAS-SF scale allowed the study group to be divided. Those who fell into the no addiction group were considered to be social media users.

**Table 2:** The participants’ Toronto Empathy Questionnaire, Toronto Alexithymia Scale, and SMAS-SF scores (N = 649).

Instruments	Mean ± SD		Min		Max
The Toronto Empathy Questionnaire	48.76 ± 5.65		18		63
The Toronto Alexithymia Scale	47.71 ± 11.49		23		83
		n		%	
Nonalexithymia (≤51 points)		419		64.56	
Possible alexithymia (52‐60 points)		139		21.42	
Alexithymia (≥61 points)		91		14.02	
SMAS-SF	73.20 ± 14.59		31		115
		n		%	
no addiction (≤ 58 points)		111		17.10	
mild addiction (59-87 points)		426		67.18	
moderate addiction (88-116 points)		102		15.72	
severe addiction (≥ 117 points)		0		0	

### 3.2 Distribution of the scale mean scores according to gender

The students’ TEQ total mean score was higher in females (P<0.001). The difference between mean scores for the TAS total was statistically insignificant between males and females. However, the DIF and EOT factors recorded statistically significant differences between the genders. The SMAS-SF total mean score was also higher in females (P=0.001). All data are presented in **Table 3**.

**Table 3:** Distribution of the scales mean scores according to the gender of the participants.

	All Mean (SD)	Range	Women Mean (SD)	Men Mean (SD)	The P-value for gender difference*
TEQ	48.76 (5.65)	18-63	49.74 (4.99)	44.97 (6.46)	<0.001
TAS total	47.71 (11.49)	23-83	47.97 (11.72)	46.68 (10.56)	0.171
DIF	17.30 (6.43)	7-34	17.75 (6.44)	15.56 (6.14)	<0.001
DDF	13.74 (4.52)	5-25	13.81(4.69)	13.47 (3.79)	0.377
EOT	16.67 (3.79)	8-28	16.41 (3.72)	17.65 (3.91)	0.001
SMAS-SF	73.20 (14.59)	31-115	74.16 (14.28)	69.45 (15.23)	0.001

*Based on the Mann–Whitney test.

### 3.3 Correlations

The DDF and EOT were negatively correlated with the TEQ; the DIF presented a much lower significant negative correlation with the TEQ. The DIF and DDF scores were positively correlated with the SMAS-SF scores, and there was no significant correlation between the TEQ and the SMAS-SF. All correlations are presented in **Table 4**.

**Table 4:** Pearson-moment product correlations between alexithymia, empathy, and social media addiction scores.

Factors	TEQ	DIF	DDF	EOT	TAS total
DIF	-0.098*	–			
DDF	-0.215**	0.624**	–		
EOT	-0.339**	0.221**	0.261**	–	
TAS total	-0.251**	0.878**	0.829**	0.555**	–
SMAS-SF	-0.0178	0.351**	0.239**	0.099*	0.323**

*P<0.05.

**P<0.001.

### 3.4 Path analysis model

Following the correlation analyses results, we performed mediation analyses to further examine the association between social media addiction, alexithymia and the degree of empathy in undergraduate medical students.

**Figure 1:**
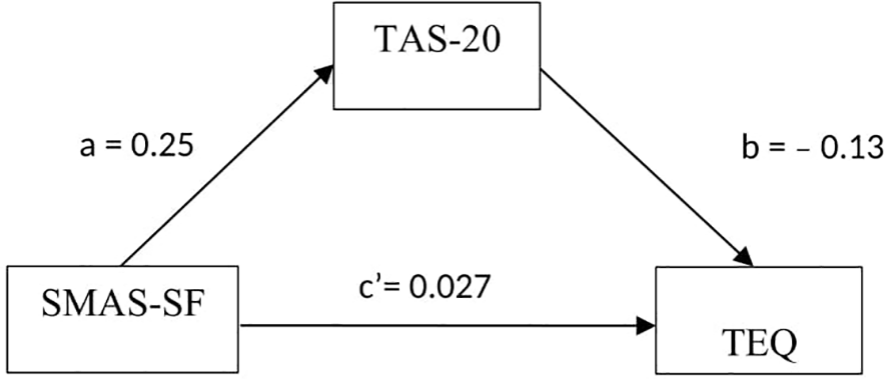
Path diagram showing alexithymia as a mediator between social media addiction and the degree of empathy in undergraduate medical students. Numbers on the single-headed arrows represent standardized regression coefficients (Beta).

In Model 1, social media addiction was not significantly associated with empathy (path c)(β= -0.0068, P =0.652). In Model 2, social media addiction had a significant relationship with alexithymia (path a)(β=0.254, P < 0.001). In Model 3, both social media addiction and alexithymia were included in the mediation model and showed a significant relationship with empathy. Simultaneously, the standardized regression coefficient (β) for social media addiction decreased from -0.007 to 0.027. Moreover, the results of the non-parametric bootstrapping method confirmed the significance of the indirect effect of social media addiction through alexithymia (95% bootstrap CI=-0.047, -0.021). A bootstrapped 95% confidence interval (CI) confirmed that the indirect effect of social media addiction had an impact of -0.034 which was produced by alexithymia as a mediator on empathy (**Table 5**). These findings corroborate our hypothesis that alexithymia may play a mediator role in the association between social media addiction and empathy. **Figure 1** illustrates the mediation model, along with standardized path coefficients.

**Table 5:** Mediating model examination by bootstrap.

	Social media addiction → Empathy			
	Effect	SE	LL 95%CI	UL 95%CI
Indirect effect	-0.034	0.006	-0.047	-0.021
Direct effect	0.027	0.015	-0.003	0.057

## 4 Discussions

In our daily life, and to function optimally as medical professionals, we employ instinct and intuition, alongside reasoning and logical deductions. In this complex inter-play, empathy plays an essential role, found at the core of the therapeutic relationship, and the essence of the doctor-patient relationship. Being able to place oneself in someone else’s place can be challenging, especially when the other is suffering. However, instrumental, as it is the only way to more precisely try to understand what that the other is going through.

The present study examined the relationships between alexithymia, social media addiction, and empathy using validated self-administered questionnaires for medical students. People with alexithymia experience difficulties understanding their feelings, as well as those of others, which could result in limitations in the empathic abilities of alexithymia individuals. On the other hand, in the workplace, they also experience difficulties in being able to socialize with colleagues, as they seem inexpressive, aloof, which, in turn, leads to a high probability of becoming unemployed (48).

There are several studies that suggest alexithymia plays an important role in the etiology of addictive behaviors (49–51). Social media is what facilitates interpersonal relationships, which can lead to addiction in people with a high level of alexithymia.

Both empathy and alexithymia are necessary qualities for someone looking to pursue a future medical career. When considering the question of the reasoning behind that, the most likely answer is that empathy allows the building of relationships, and facilitates understanding what patients think, thus making it easier for healthcare workers to respond appropriately. Empathy allows us to manage what we feel, even when faced with stressful situations, without being overwhelmed (52). Moreover, empathy is what allows us to offer unconditional and disinterested help to another.

In our study, the total TEQ score was 48.76 ± 5.65, which is slightly higher than those reported in the literature. Differences between women and men were also significant, favoring the former. A recent study carried out in 57 countries, in 2022, reached the same conclusions, namely that women, regardless of their age and country of origin, scored higher than men in the “Reading the Mind in the Eyes” test, used on a broad scale, to measure the degree of “cognitive empathy” (53).

In this study, the prevalence of medical students with alexithymia was of 14.01%, slightly higher than that reported in the general population (13, 53), but lower than those reported in other student populations (54, 55). For someone with alexithymia, understanding one’s own emotional issues is problematic, which is even more difficult when dealing with someone else. An alexithymic health professional will struggle in their interactions, ultimately negatively impact the therapeutic alliance and treatment they offer others (56). Therefore, we suggest that medical students and health professionals in general should be familiar with the concept of alexithymia and its significance in their own personal and professional lives (57, 58).

Among other results, we also found a negative, and significant correlation between the TEQ Scale and the mean TAS-20 scores. Current research supports a cerebral connection between alexithymia and empathy, with the connection established at the level of the insular cortex, where the processing of internal affective states is carried out. The anterior cingulate cortex is associated with emotion processing and social rewards, and a reduced activity in this cerebral area may be associated with alexithymia (59). In this view, those with alexithymic traits will tend to have less empathy. Consequently, young people undergoing healthcare education should determine their own capacity for empathy, and those who struggle in this area should receive specific psycho-education on empathy (60).

In our study, we chose to position the personality trait as a mediator rather than a predictor due to specific theoretical considerations. While personality traits are generally considered stable and develop early in life, recent research suggests that they can also be influenced and shaped by environmental factors and behaviors acquired later in life, such as social media use. For instance, there is evidence that frequent engagement in certain behaviors can reinforce or even modify certain personality traits over time, suggesting a bidirectional relationship (61, 62). Valdespino et al. (63) have explored the temporal relationship between alexithymia and empathy, demonstrating that alexithymia serves as a precursor to empathy abnormalities. Therefore, in our theoretical model, social media use is posited as an influential factor that could shape personality traits, thereby justifying its role as a predictor.

Our path analysis model showed that alexithymia is an essential mediator between social media addiction and empathy. Empathy implies one’s capacity to adopt and understand another’s experiences and emotions. Secondary to their difficulty in identifying and describing emotions, people with alexithymia might find it strenuous to imagine and perceive the emotional experiences of others. In terms of virtual communication, social media rarely offers non-verbal clues, such as facial expressions, body language and tone of voice, essential elements on which communication skills are built and how we, as a species, have learned to understand emotional states in others. Also, social media usually offers curated, unrealistic portrayals of life, as well as acts of aggression, which some-times surmount current constrains and policies, subsequently being responsible for traumatic effects on their consumers. Thus, this confirms studies showing that maltreatment and trauma may be a contributing factor in alexithymia (64–66).

For people with alexithymia, who struggle on a daily-basis to accurately interpret said clues, the interposition of social media raises their inaccurate interpretation of other people’s emotions, and lowers their empathy. Increasing the overall time spent on social media might impair empathic responses and reduce the quality of social interactions. According to certain clinical studies, people suffering from social media addiction also have lower empathy levels (67, 68), adding to the well-established theory that empathy correlates with social relationships (69).

Although the present study shows that alexithymia can mediate the relationship between social media use and empathy, it is not the major determining factor, since there are specific individual coping mechanisms, and characteristic personality traits, which can also influence the dynamics of this relationship.

The present research has several limitations, the main one being that it was con-ducted in a single location, on a population with shared cultural traits. Although alexithymia is a mediator of empathy, only about 11% of the variability in empathy is explained by this model. Since the SRMR was zero, other factors should be considered in the future. The study was performed on medical students, so conclusions cannot be extended to the general population. However, the number of participants in our study was large enough to consider the results as reliable. For future studies, objective measures and investigator ratings should be added to the assessment of the connections between alexithymia and empathy.

A significant limitation of this study is the use of cross-sectional data for mediation analysis. While we identified a potential mediating role of alexithymia in the relationship between social media addiction and empathy, it is important to acknowledge that cross-sectional designs limit our ability to infer causality or the temporal order of these relationships. Longitudinal data would be necessary to confirm these mediation effects over time and to establish a clearer causal pathway.

## 5 Conclusions

According to our best knowledge, this is the first paper that analyzes alexithymia as a mediating factor between social media addiction and empathy. Our results indicated a significant negative correlation between empathy and alexithymia in medical students. Alexithymia was a mediator between social media addiction and empathy. Therefore, we recommend timely and specific efforts to identify levels of alexithymia in more medical students, which can lead to the design of tailored interventions aimed at increasing emotional awareness and aptitudes.

## Data Availability

The original contributions presented in the study are included in the article/supplementary material. Further inquiries can be directed to the corresponding author.
